# Assessing the Clinical Utility of Expanded Macular OCTs Using Machine Learning

**DOI:** 10.1167/tvst.10.6.32

**Published:** 2021-05-26

**Authors:** Andrew C. Lin, Cecilia S. Lee, Marian Blazes, Aaron Y. Lee, Michael B. Gorin

**Affiliations:** 1 Department of Ophthalmology, School of Medicine, University of Washington, Seattle, WA, USA; 2 Department of Ophthalmology, New York University, New York, NY, USA; 3 Department of Ophthalmology, University of California, Los Angeles, CA, USA

**Keywords:** machine learning, primary open-angle glaucoma, optical coherence tomography, age-related macular degeneration, diabetic macular edema

## Abstract

**Purpose:**

Optical coherence tomography (OCT) is widely used in the management of retinal pathologies, including age-related macular degeneration (AMD), diabetic macular edema (DME), and primary open-angle glaucoma (POAG). We used machine learning techniques to understand diagnostic performance gains from expanding macular OCT B-scans compared with foveal-only OCT B-scans for these conditions.

**Methods:**

Electronic medical records were extracted to obtain 61 B-scans per eye from patients with AMD, diabetic retinopathy, or POAG. We constructed deep neural networks and random forest ensembles and generated area under the receiver operating characteristic (AUROC) and area under the precision recall (AUPR) curves.

**Results:**

After extracting 630,000 OCT images, we achieved improved AUROC and AUPR curves when comparing the central image (one B-scan) to all images (61 B-scans). The AUROC and AUPR points of diminishing return for diagnostic accuracy for macular OCT coverage were found to be within 2.75 to 4.00 mm (14–19 B-scans), 4.25 to 4.50 mm (20–21 B-scans), and 4.50 to 6.25 mm (21–28 B-scans) for AMD, DME, and POAG, respectively. All models with >0.25 mm of coverage had statistically significantly improved AUROC/AUPR curves for all diseases (*P* < 0.05).

**Conclusions:**

Systematically expanded macular coverage models demonstrated significant differences in total macular coverage required for improved diagnostic accuracy, with the largest macular area being relevant in POAG followed by DME and then AMD. These findings support our hypothesis that the extent of macular coverage by OCT imaging in the clinical setting, for any of the three major disorders, has a measurable impact on the functionality of artificial intelligence decision support.

**Translational Relevance:**

We used machine learning techniques to improve OCT imaging standards for common retinal disease diagnoses.

## Introduction

Since optical coherence tomography (OCT) was first described in 1991,^1^ it has revolutionized the clinical management of various retinal pathologies, and its application continues to accelerate at a tremendous pace.^2^ OCT can generate three-dimensional (3D) models of the retina at a submicrometer resolution and has become the standard of care for evaluating many ophthalmic disorders,^3^ such as age-related macular degeneration (AMD)^4^^,^^5^ and diabetic macular edema (DME).^6^^,^^7^ Although the diagnosis of primary open-angle glaucoma (POAG) has been based on the assessment of structural and functional damage identified through the use of fundus photography and visual fields, respectively, there is an increasing use of macular OCT thickness evaluation as a reliable metric for glaucoma disease detection and longitudinal management.^8^^,^^9^ These conditions remain the three most common retinal causes of blindness and low vision in the United States and worldwide.^10^ Thus, the use of OCT imaging in these conditions is profound and continues to expand.

Despite significant advancements in OCT technology and usage, clinical imaging protocols for POAG, AMD, and DME have not progressed at a similar pace. There are no standardized imaging protocols or guidelines for the evaluation of these highly prevalent diseases; thus, clinicians are left evaluating a varying number of OCT scans. Several studies have shown that different areas of the macula may be important for detecting subtle, early abnormalities of glaucoma, AMD, and DME.^9^^,^^11^^,^^12^ However, there is no objective evaluation on whether the expanded OCT coverage is beneficial in detecting these diseases to date. Knowing the optimal macular OCT coverage would be critical in routine clinical practice to ensure that no crucial information is missed in evaluating diseases.

Recently, OCT evaluation has been dramatically enhanced by the introduction of artificial intelligence (AI). Machine learning approaches have been successfully applied to diagnose, segment, and prognose several retinal pathologies, including diabetic retinopathy,^13^^–^^16^ DME,^17^^–^^19^ AMD,^2^^,^^20^^,^^21^ and POAG,^22^^–^^24^ providing novel, automated methods for unbiased evaluation of various pathologies. Algorithms can also objectively assess the added benefit of each incremental macular coverage in the diagnostic accuracy of major conditions. In clinical settings, the same instruments are often used with a range of scan areas and scan line densities. Often, this is driven by the default settings of the machines and/or by the need for rapid image acquisition and review. Increasingly, clinicians are interested in using these clinical scans to both train and diagnose clinical disorders and predict progression risk. To our knowledge, the diagnostic value of broader anatomic coverage has not been systematically evaluated in the literature. We sought to employ an ensemble machine learning approach to objectively evaluate the clinical utility of expanding OCT macula coverage with regard to disease classification accuracy using a dataset composed of patients with AMD, DME, and/or POAG. We chose to use the binary distinction of disease classification, labeled as affected or unaffected, as a proxy of the global information content of these scans, rather than focusing on the identification or quantification of specific retinal features, as this is more reflective of the use of neural networks to evaluate among subtle disease distinctions. We included POAG with the two other retinal conditions due to its prevalence and specific impact on the various cellular layers and features of the retina when compared with AMD or DME.

There is considerable variability among clinicians in selecting the area of coverage and density of scans. Although smaller areas and less dense scans are convenient for efficiency of acquisition and review, we hypothesize that one potentially sacrifices valuable information that would inform a clinician as well as an AI algorithm. It is likely that the impact could differentially affect the utility of OCTs.

## Methods

### Study Design

This retrospective database study was approved by the institutional review board at the University of Washington and adhered to the tenets of the Declaration of Helsinki. We used de-identified patient data in accordance with the Health Insurance Portability and Accountability Act of 1996 Privacy Rule. In the following paragraphs, we describe the ensemble approach used in this study, which involved combining a convolutional neural network (CNN) with a random forest classifier.

We obtained all OCT images and clinical data of patients seen in the Department of Ophthalmology at the University of Washington Medical Center (between the years of 2006 and 2019). Macular OCT scans were extracted from the Heidelberg Spectralis (Heidelberg Engineering, Heidelberg, Germany) imaging database. Using a de-identified clinical data repository, we extracted patients’ demographic and clinical information from electronic medical records. International Classification of Diseases Ninth and Tenth Revision codes were used to include all patients with AMD, DME, and POAG (Supplementary Figure S1 and Table S1). The dataset was randomly split into training, validation, and held-out test sets based on unique patient identifiers into a 60:20:20 ratio. Thus, each data subset contained all corresponding visits associated with a particular patient without overlap.

Of note, when a patient had been diagnosed with AMD, all subsequent visits were labeled with a diagnosis of AMD in addition to any concurrent diagnosis of DME and/or POAG. Thus, a single patient visit could be labeled with all three diagnoses simultaneously. The method of forward propagation used for AMD patients was not applied to those patients diagnosed with DME or POAG. Each raster macular OCT was extracted as an individual image, with up to 61 images per eye per visit per patient included. All OCT images selected were B-scans of size 768 × 496-pixel resolutions. The contiguous B-scans were taken 0.125 mm apart for a total anatomic coverage of 7.50 mm across a complete 61-image set.

### VGG-BN-16 Convolution Neural Network

The original OCT images were extracted from the database in PNG format. Images were not processed to remove noise or to enhance contrast for generalizability purposes. All images were center cropped to a size of 496 × 496-pixel resolution. The images were resized to 299 × 299 pixels for input to the neural network while maintaining the same height-to-width aspect ratio. To combat overfitting, we applied the following data augmentation techniques to each training image before it was fed into the deep learning model: random shuffling, random rotation up to 15 degrees, random shearing up to 10% of the image size, random width shift up to 10% of the image width, and random height shift up to 10% of the image height. A modified version of the pre-trained VGG-BN-16 CNN was used via a transfer learning approach with ImageNET.^25^ All layers and weights were unfixed and retrainable. The top layers were removed and replaced with a 256-neuron fully connected layer with a ReLu activation function, followed by a dropout layer of 0.2 and finally a three-neuron fully connected layer with a sigmoid activation function that outputted probabilities from binary cross-entropy losses for each of the three diagnoses independently. The model was compiled and trained using the sum of the three separate cross-entropy losses. The VGG-BN-16 network was trained to classify OCT-level disease diagnoses for each of the three diseases separately for each B-scan of an OCT scan. Thus, in an OCT scan with 61 B-scans, the network outputted 61 separate sets of diagnoses, with each diagnosis uninfluenced by other B-scans in the set. The output data of the network was later concatenated prior to serving as input data for the random forest model. Adam optimizer was used with the learning rate set to 1e–4, and a batch size of 16 was implemented.

Using the training and validation datasets only, training was performed for 50 epochs with a patience of 10 epochs, and the best model was saved based on the lowest validation loss obtained. Each image from the data subsets was fed into its respective model for feature map extraction, with the best weights frozen for a two-dimensional tensor output of size 256 × 1.

**Figure 1.**
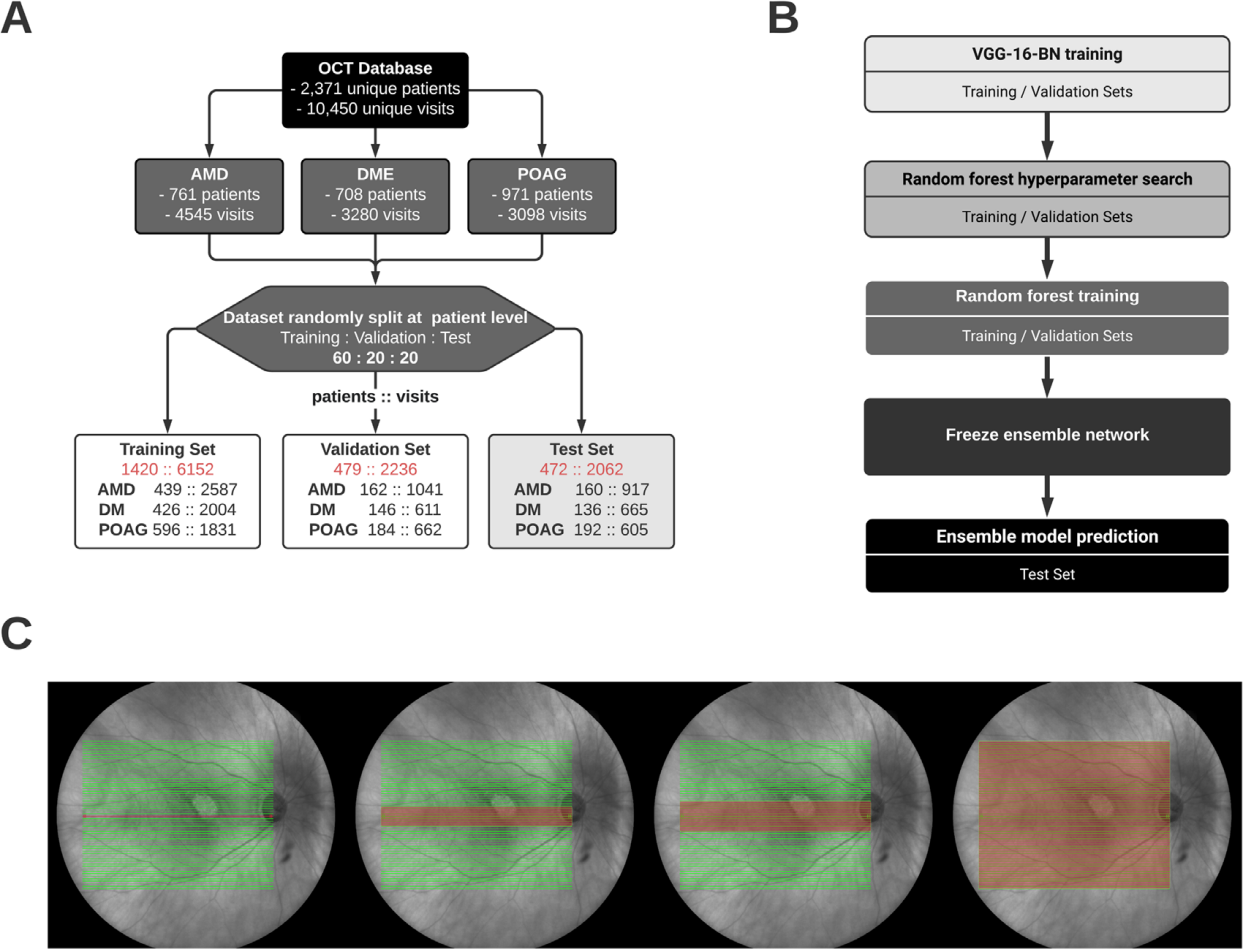
Schematic of data pre-processing (**A**), stages of model development (**B**), and areas of coverage (**C**). The dataset was randomly split at the patient level 60:20:20 for training, validation, and testing sets. The training and validation datasets were used for training of the VGG-BN-16 neural network, as well as training and hyperparameter search of the random forest model. The held-out test set was only introduced for prediction after the best models were frozen. Each subsequent model had increasing areas of coverage (**C**), with models 1 (one B-scan), 5 (nine B-scans), 7 (13 B-scans), and 31 (61 B-scans) shown.

All models were fed imaging data from OCT scans of the same 768 × 496-pixel image size, each with 61 raster lines. Consequently, all images maintained the same raster line density with 0.125 mm of macular area between adjacent B-scans. The first model was trained and evaluated on the central foveal B-scan only, which served as the baseline. In order to evaluate the impact of information gain from additional non-foveal scans, each subsequent and independent model incorporated two vertically adjacent peripheral B-scans, increasing the anatomic coverage by 0.25 mm in the vertical direction. This method was applied until all 61 images from one patient visit were used in the final model, for a total of 31 independently trained models (Fig. 1). As such, the 31st model covered the full 7.50 mm. This method of gradually expanding the vertical area of coverage while maintaining the same horizontal area of coverage provided a systematic approach for objectively quantifying the change in gain of diagnostic information from increasingly peripheral B-scans beyond the fovea.

For patient visits without 61 total images, all of the available images that satisfied a full image-set were used. If an adjacent image was unavailable for the subsequent model, the entire patient visit was removed from the dataset. Thus, a patient visit with only three B-scans would be included for training and evaluation for the first and second models but would not be included in the third model.

### Random Forest

To combine predictions across B-scans, we employed a random forest ensemble approach that utilized the feature vectors extracted from each of the 31 independently trained CNNs. Of note, 3D CNNs for volumetric space prediction were impractical, as the spacing between adjacent B-scans made model construction unfeasible. The 61-raster-scan pattern spacing in these OCTs was sufficiently wide such that a convolutional operation in the third axis would not result in substantive interpretation. In addition, precise registration between the B-scans was not readily achievable to make 3D convolutions meaningful. For each model, the 256 × 1 feature vector obtained from the central foveal B-scan was concatenated with the feature vectors of the adjacent B-scans (excluding the baseline model) and padded equally on either side with value of 0.0 until a vector of size 15,616 × 1 was reached. As a result, all datasets evaluated by the random forest model were of equal dimensions, and the final eye-level diagnoses were influenced by all of the B-scans within the same OCT set. We then trained 31 independent random forest classifiers with the same training and validation sets used in the VGG-BN-16 networks, again with the best models saved. Hyperparameter tuning was obtained through randomized search using fivefold cross-validation. Selected hyperparameters gave the best cross-validation score. The random forest models returned probability estimates on each of the three diseases, with values between 0 and 1, allowing for multiple diseases to be present in the same eye. Only at the end were the held-out test datasets used to evaluate model performance and report performance metrics.

### Statistical Analysis

To reduce variability due to random initialization and local minima, the ensemble network was run 10 times per model with random seeds for a total of 310 generated models, including the baseline model. The final area under the receiver operator characteristics (AUROC) curve and area under the precision recall (AUPR) curve for each of the three diseases were individually calculated and averaged. The standard error for each of the metrics per model was calculated across the 10 runs. All models were evaluated against the baseline model for significance by a one-tailed two-sample Wilcoxon rank sum with a Bonferroni correction of 180 (30 models × 3 diseases × 2 AUCs). A *P* value of <0.05 after correction was considered significant.

### Implementation

The CNN used for training and evaluation of the models was written in Python 3.7.1 (http://www.python.org) and implemented in Keras 2.2.4 (https://keras.io/) with TensorFlow 1.4.1 (https://www.tensorflow.org/) as the backend. Tools including Pandas, NumPy, Scikit-learn, matplotlib, and GNU-Parallel were used for data processing and stratification, random forest analysis, and construction of the ROC and PR curves. The code has been open sourced and is available on GitHub (https://github.com/uw-biomedical-ml/Expanded-MacOCT).

## Results

**Figure 2.**
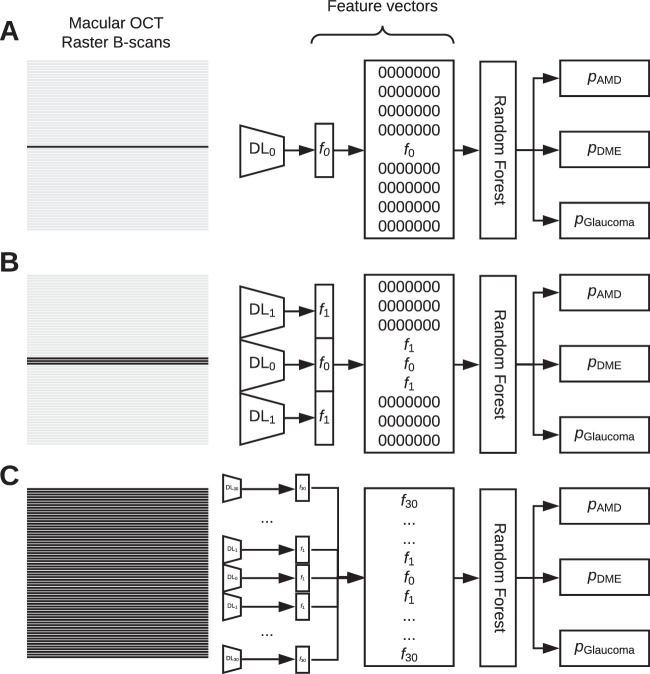
Schematic of ensemble network construction of the first (**A**), second (**B**), and last (**C**) models. For the first model (**A**), the central foveal B-scan was used to pre-train a VGG-BN-16 network for extraction of one 256 × 1 feature vector which was 0 padded equally on both sides and introduced to the random forest model for diagnostic prediction. For the second model (**B**), three B-scans that included the central foveal B-scan and two immediately adjacent scans were used to pre-train a separate VGG-BN-16 model for extraction of three 256 × 1 feature vectors. These feature vectors were concatenated and 0 padded equally on both sides and introduced to a separate random forest model for diagnostic prediction. For the final model (**C**), 61 B-scans were used to pre-train the last VGG-BN-16 network for the extraction of 61 256 × 1 feature vectors. These outputs were concatenated and introduced to a final random forest model for diagnostic prediction.

A total of 630,000 OCT images from 2371 patients across 10,450 patient visits were extracted. The images were split into training, validation, and held-out test sets at the patient level as described in Figure 1. The baseline demographic factors for the study population are shown in the Table. Each of the 31 ensemble networks was constructed based on the methods described in Figure 2. An end-to-end deep learning approach was attempted but led to inferior performance compared to the random forest ensemble. All training was performed by a pre-trained VGG-BN-16 network for 50 epochs with a patience of 10 epochs, and the best model was saved based on the lowest validation loss obtained (Supplementary Fig. S2). All images were fed again into a saved best model for feature extraction at the image level. For the first model, feature vector concatenation was not performed, as there were no adjacent images used. For the 31st and final model, 0.0 padding was not performed, as the concatenated vectors resulted in the maximum size of 15,616 × 1. Each ensemble network was run 10 times based on randomly set seed values. Incomplete OCT scans were removed from the dataset as per the methods described previously.

**Table.** Baseline Demographic Factors at the Patient Level

	AMD	DME	POAG	Total
Patients, *n*	761	708	971	2371
Age (y), mean (SD)	78.0 (8.3)	59.4 (12.1)	69.8 (11.6)	69.9 (13.1)
Gender, *n* (%)				
Male	307 (40.3)	396 (55.9)	533 (54.9)	1172 (49.4)
Female	454 (59.7)	312 (44.1)	438 (45.1)	1199 (50.6)
Race, *n* (%)				
American Indian or Alaska Native	9 (1.2)	15 (2.1)	12 (1.2)	36 (1.5)
Black/African American	29 (3.8)	127 (17.9)	190 (19.6)	339 (14.3)
Asian	114 (14.9)	125 (17.7)	145 (14.9)	380 (16.0)
Native Hawaiian or Pacific Islander	2 (0.26)	41 (5.8)	6 (0.62)	49 (2.1)
White	577 (75.8)	357 (50.4)	571 (58.8)	1448 (61.1)
Declined/unknown	30 (3.9)	43 (6.1)	47 (4.8)	119 (5.0)
OCT macular volume (mm^3^), mean (SD)	16.7 (0.89)	16.8 (1.02)	16.9 (1.01)	16.8 (0.95)

**Figure 3.**
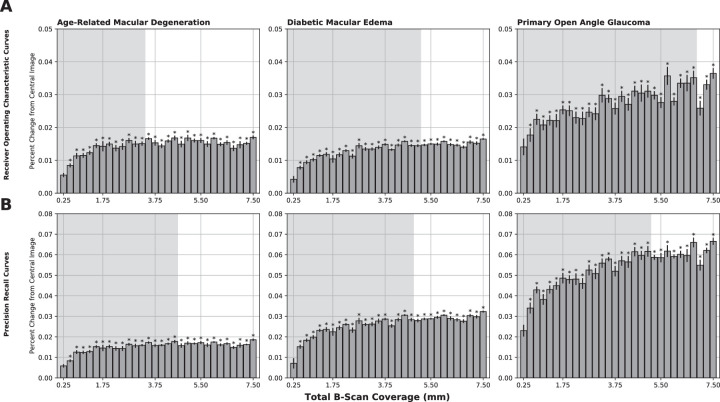
Percent change in AUROC (**A**) and AUPR (**B**) curves as a function of increasing total B-scan coverage across all three diseases. Error bars indicate the standard error calculated from each 10-run set. Each *shaded area* represents the range of information gain prior to the point of diminishing returns. All asterisks (*) indicate *P* < 0.05 after a Bonferroni correction of 180.

We achieved AUROC curves of 0.9556, 0.9735, and 0.8887 and AUPR curves of 0.9514, 0.9530, and 0.8203 from the central foveal images for AMD, DME, and POAG, respectively. We achieved AUROC curves of 0.9718, 0.9895, and 0.9211 and AUPR curves of 0.9691, 0.9838, and 0.8749 from the full 7.50-mm coverage for AMD, DME, and POAG, respectively. A plot of the percent change in the calculated AUROC and AUPR curves of each model with respect to the first model (Fig. 3) revealed varying trends in information gain and points of diminishing return among the three diseases. The AUROC and AUPR values for each model were individually normalized to 0% and 100% by the first and last model, respectively. The point of diminishing return was found by applying a running three-model consecutive average with a threshold set as ≥90% of the maximum gain as obtained from the 31st model. The point of diminishing return is reflected in Figure 3 by the border of the gray-shaded background. AMD and DME were found to have the most gain in AUROC performance from the central 2.75 mm (14 B-scans) and 4.50 mm (21 B-scans) of coverage before diminishing returns, respectively, whereas POAG had continued information gain up to 6.25 mm (28 B-scans) of volume coverage. The results for AUPR followed a similar trend, with AMD, DME, and POAG having information gains up to 4.00 mm (19 B-scans), 4.25 mm (20 B-scans), and 4.50 mm (21 B-scans), respectively, before diminishing returns. Taking into account the Bonferroni correction of 180, all models except for the 0.25-mm (three B-scans) model, were found to have significantly higher AUROC and AUPR values for all three disease states.

**Figure 4.**
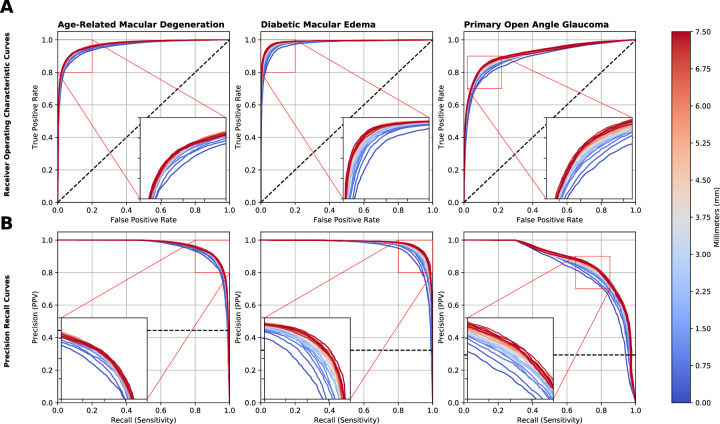
Aggregated ROC (**A**) and PR (**B**) curves with respect to variable B-scan areas of coverage across all three diseases. The *blue* and *red* color scale indicates least and most macular area of coverage, respectively. An inset graph is shown for ease of differentiation.

Figure 4 demonstrates by colormap the ROC and PR curves of the 31 models as a function of increasing B-scan coverage on a scale of blue to red, with blue representing the central foveal slice only with 0.00-mm coverage and red representing the full 61 set with 7.50-mm coverage. Across all models, the overall trend demonstrated increases in both AUROC and AUPR curves as macular coverage increased from 0.00 mm (one B-scan) to 7.50 mm (61 B-scans).

## Discussion

Using over 630,000 OCT images from over 2300 patients, we have identified that all three diseases (AMD, DME, and POAG) benefit from the evaluation of non-central OCT B-scans. Based on AUROC and AUPR values, the most gain of information for the diagnosis originated from the following range of macular coverage: 2.75 to 4.00 mm in AMD, 4.25 to 4.50 mm in DME, and 4.50 to 6.25 mm in POAG. Our results revealed the earliest point of diminishing return in AMD requiring the least total macular coverage, followed by DME and finally POAG. Furthermore, compared with images labeled with POAG, the AMD and DME cohorts were found to have greater initial AUROC and AUPR curves obtained from the central foveal image alone and smaller percent increases with respect to expanded macular coverage.

Previous studies support the results of our systematic evaluation. Studies of early glaucoma have demonstrated abnormal thinning of the retinal nerve fiber layers in both the macular and perimacular regions,^9^^,^^11^^,^^12^ and glaucomatous damage to this critical region is overlooked without full macular scans.^26^^,^^27^ In AMD, early manifestations are often detected beyond the central fovea, with atrophic patches typically occurring in the parafoveal retina before central progression with associated dramatic visual loss.^28^^,^^29^ Rudolf et al.^30^ found regional differences in the morphology and composition of drusen between the macular and extramacular regions, defined as a radius less than or greater than 3.00 mm from the fovea, respectively. A recent study specifically addressed this lack of clarity in the clinical significance of peripheral OCT coverage by evaluating cohorts of AMD and normal patients and found associations between AMD and perimacular drusen found in macular OCT.^31^ Studies examining scans beyond the macula have demonstrated that peripheral retina changes, although less symptomatic, were found to be significantly correlated with DME.^32^

In AMD and DME, a cross-sectional B-scan of the central retina is a major contributor to the clinician's diagnosis and management plans. This is evidenced by numerous OCT deep learning studies with models based on the central foveal B-scan only,^33^^,^^34^ an averaged image of all scans,^35^ or a subset of the full-scan centered around the fovea.^2^ In contrast, deep learning studies of glaucomatous patients who have relatively more peripheral OCT abnormalities typically utilize the full B-scan image set to create macular thickness maps of nerve fiber layers^36^ or to ascertain complete volumetric data.^37^^,^^38^ Although changes in peripheral structures due to POAG are more common and more easily detected on examination, AMD and DME have been shown to exhibit early subtle peripheral changes.^32^^,^^39^ In these earlier stages of AMD and DME, extrafoveal regions hold critical information for early detection or disease progression prior to clinically apparent deterioration.

The fovea and macula are only 2.00 and 6.00 mm in diameter, respectively. Within the macula, the fovea contains the highest density of cone photoreceptors and is the only region of the retina where the visual acuity can reach 20/20. Together, this small region accounts for nearly 10% of the entire visual field.^40^ Consequently, pathologic changes or lesions to this region more often impact central visual function; however, the pathologic changes that occur outside the fovea may contain relevant features for the diagnosis. The initial AUROC and AUPR curves and relative trends in information gain for the diagnostic accuracy of three conditions were within the context of their respective pathophysiologies.

It is notable to differentiate the information gain from extending the macular coverage in diagnosing POAG from AMD and DME. The majority of retinal changes attributed to AMD typically occur at or near the fovea, predominantly impacting central vision. This is supported by the point of diminishing return result obtained by the model, which was found to be at 2.75 mm of coverage, or just beyond the foveal region. Thus, although it is important to image the entirety of this region for the evaluation of AMD, further expanded imaging offers diminishing returns. The DME-labeled images showed a similar trend, with the point of diminishing return found to be at 4.50 mm of coverage. The standard for DME by the Early Treatment Diabetic Retinopathy Study and in clinical trials only includes the area within 0.50 mm of the fovea. However, multiple studies have demonstrated extrafoveal changes within 6.00 mm of the fovea that correlated with DME.^41^^,^^42^ Our results are consistent with these findings and support obtaining a greater area of macular coverage than suggested for AMD.

In contrast, there was almost no point of diminishing return for POAG, with up to 6.75 mm of coverage being necessary before the onset of marginal returns. POAG typically exhibits greater peri- and parafoveal structural changes with associated peripheral vision loss. Even with full macular coverage, the OCT covers only a fraction of the optic nerve fibers; thus, some arcuate defects may only be visible at the very peripheral OCT scans of the macula. Our findings were consistent with the known pathology, with relatively lower initial AUROC and AUPR curves and a greater trend in coverage-based information gain compared with AMD and DME.

Our study results have important clinical implications. Some OCT devices allow clinicians to choose the extent of macular coverage for OCT imaging, and these thresholds should be carefully selected based on the specific disease being assessed. Previous literature has shown that glaucoma specialists do not take full advantage of comprehensive OCT scanning and often only obtain scans of the optic disc, but our results highlight that evaluation of full macular area would increase the diagnostic accuracy.^26^^,^^27^ Furthermore, some home OCT devices are designed to only image the central 1.0 mm for AMD, which would provide insufficient evaluation according to the results of this study. It is also important to recognize that regardless of the differences in marginal return, expanded coverage appreciably improves the classification performance of all retinopathies studied. Future studies addressing whether the additional macular coverage also increases the prediction of treatment response or overall prognosis of the diseases in addition to the diagnosis would be helpful.

If we intend to use OCT images as an input for AI algorithms, we need to recognize that predictive algorithms will work better for some disorders over others and that the extent of coverage of the macula may have differential impact. However, it is heartening to note that for all three studied conditions, we demonstrated improved benefit from more extensive macular coverage. This also suggests that features that lie outside of the critical central area of the macula can inform a neural network. It is reasonable to conclude that these benefits will extend to other types of analyses of disease states that were not assessed in this initial study, such as the risk and/or rate of disease progression.

This study has several limitations. We included only scans of a specific size from patients diagnosed with AMD, DME, or POAG at a single academic center with an ensemble network trained only on these images; however, we did not exclude any patients or images with poor quality. Although the external generalizability is unknown, the goal of the study was to examine the clinical utility of expanded raster scan patterns in different diseases rather than to train an automated diagnostic model. For the same reasons, we chose not to include healthy eyes. In addition, we were not able to provide visualizations of the deep learning model because of the random forest method used to ensemble the predictions. Beyond the two-dimensional CNN and random forest networks that we employed, newer techniques could be further investigated as additional ways to combine data from OCT B-scans. These methods include spatiotemporal models that are designed to incorporate data from adjacent and linked images, including recurrent neural networks, specifically long short-term memory models, and 3D CNNs. Long short-term memory networks contain cycles that feed back into the network information from prior time steps as inputs to influence future predictions in subsequent time steps. In principle, these networks hold long-term temporal contextual information, allowing the exploitation of a dynamically changing window over the totality of the input sequence. Similarly, 3D convolutions have theoretically greater spatiotemporal learning abilities that could be effectively applied to OCT B-scans with greater density.

As the spectrum of disease severity encompasses unique and varying phenotypic features for each of the pathologies, model detection and scoring of these parameters are undoubtedly affected by the standardized areas we delineated for data processing. A feature-based approach of training the models using predefined labels with discrete criteria for disease severity could provide insight into the specific image areas used in disease determination. However, there were several considerations that led us to a binary approach instead, in which the networks themselves learned to extract features to determine whether an image was AMD or not AMD, DME or not DME, and POAG or not POAG. First, we did not have the access or the capability to manually label and curate such a large database (630,000 images). Second, by training the models in this agnostic fashion, the networks learned to self-extract features for determination of disease likelihood, which has been shown to be more powerful than restricting the model with human-influenced frameworks of severity progression.^43^^,^^44^ Finally, the carefully balanced datasets allowed the network an equal opportunity to extract and learn the features associated with each disease across all disease severities simultaneously without externally introduced bias. Thus, although the results do not allow for insight into the regions of interest identified by the models, they objectively measure the information gain as a function of increased area of coverage provided by additional B-scans.

In conclusion, we constructed a series of machine learning ensemble networks that objectively demonstrated increased classification accuracy of AMD, DME, and POAG that correlated with increasing macular coverage. We found significant differences in the amount of coverage area required for diagnostic accuracy between the three diseases, with the largest macular area being relevant in POAG followed by DME then AMD. For the evaluation of AMD, DME, and POAG, our study suggests that the extent of macular coverage by OCT imaging should be disease specific. Under such recommendations, sufficiently broad macular OCTs may improve the accuracy of disease recognition and assessments of severity and/or progression by either clinicians or AI systems.

## Supplementary Material

Supplement 1

Supplement 2

Supplement 3
