# Supplementary material for: Assessing the Clinical Utility of Expanded Macular OCTs Using Machine Learning
Source: Transl Vis Sci Technol. 2021 May 26;10(6):32. doi: 10.1167/tvst.10.6.32 (PMC8161701; doi:10.1167/tvst.10.6.32)
Supplement: Supplement 1 [file tvst-10-6-32_s001.pdf]

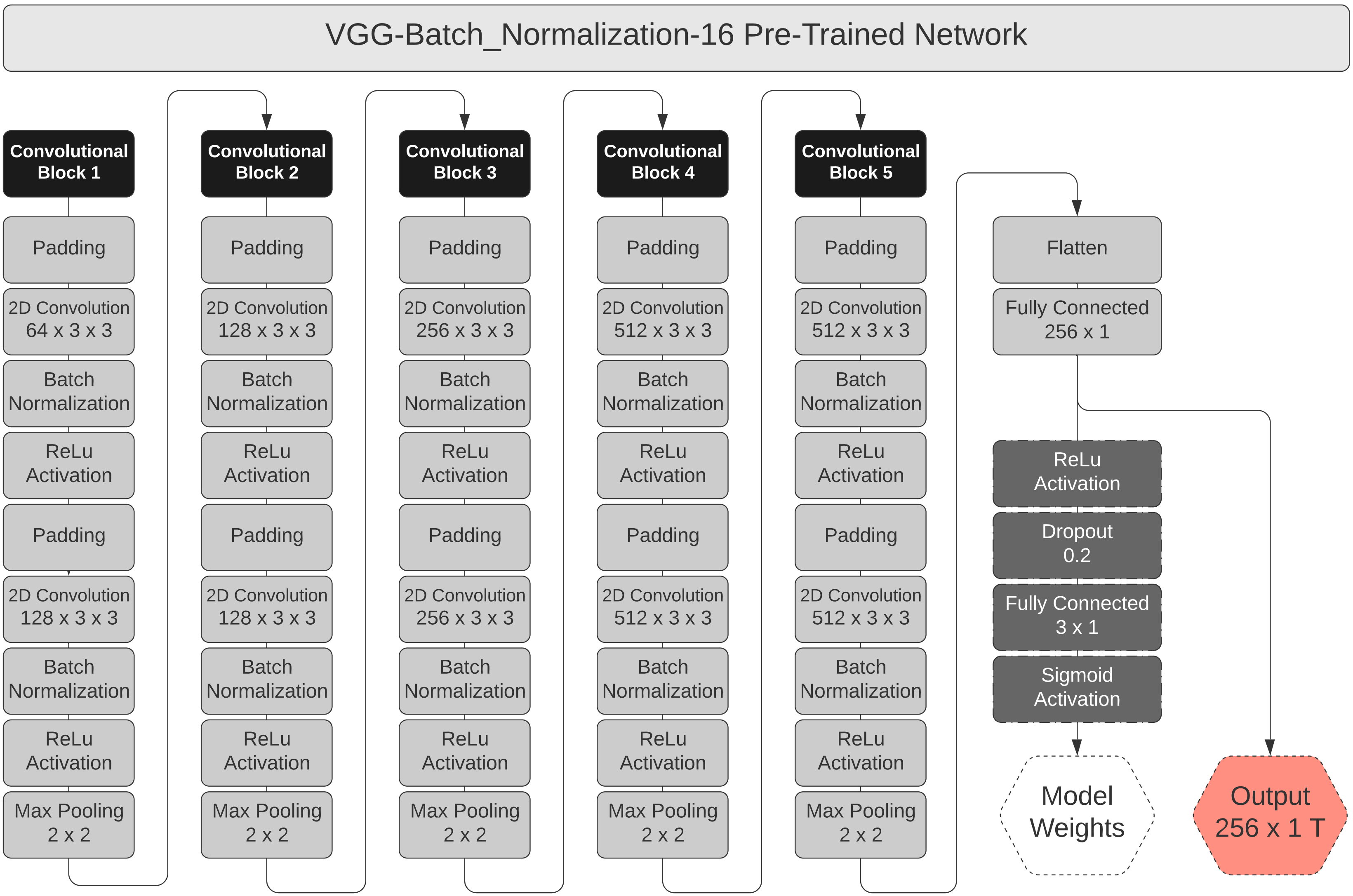

Supplemental Figure 1: Schematic of VGG-BN-16 network. Images are fed into the pre-trained network and the best model is saved by lowest validation loss. The images are re-fed back into a frozen model with best weights. The final output of each image is an intermediate layer tensor of size 256 x 1.
