# Supplementary material for: Assessing the Clinical Utility of Expanded Macular OCTs Using Machine Learning
Source: Transl Vis Sci Technol. 2021 May 26;10(6):32. doi: 10.1167/tvst.10.6.32 (PMC8161701; doi:10.1167/tvst.10.6.32)
Supplement: Supplement 2 [file tvst-10-6-32_s002.pdf]

A

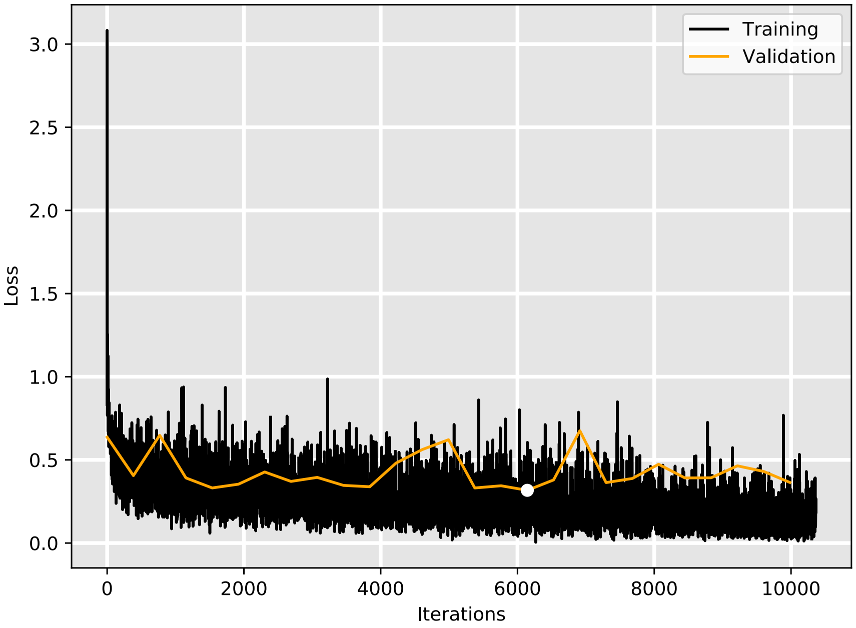

B

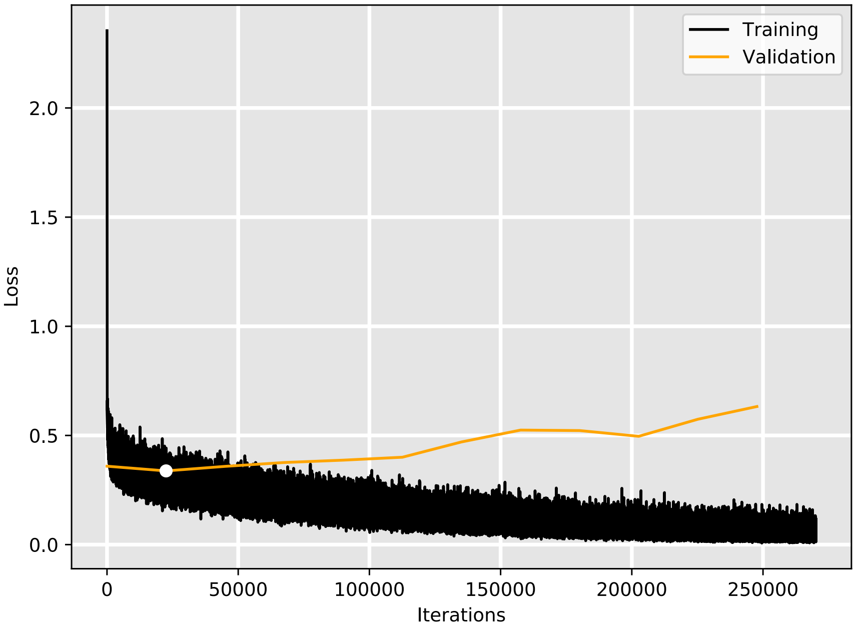

Supplemental Figure 2: Training and validation losses of VGG-BN-16 network. The training and validation losses for the central foveal B-scan **(A)** and the full 61 B-scan set **(B)** are illustrated with the lowest validation loss designated by the white circle.
