# Supplementary material for: Assessing the Clinical Utility of Expanded Macular OCTs Using Machine Learning
Source: Transl Vis Sci Technol. 2021 May 26;10(6):32. doi: 10.1167/tvst.10.6.32 (PMC8161701; doi:10.1167/tvst.10.6.32)
Supplement: Supplement 3 [file tvst-10-6-32_s003.pdf]

Supplemental Table 1: **Selected ICD 9 and 10 codes for dataset collection.**

Diabetic Macular Edema	Age-Related Macular Degeneration	Primary Open-Angle Glaucoma
E11.311x	H35.31xx	H40.11xx
E11.321x	H35.32xx	365.11
E11.331x	362.50	
E11.341x	362.51	
E11.351x	362.52	
E11.311x		
E10.321x		
E10.331x		
E10.341x		
E10.351x		
362.07		
